# Supplementary material for: Pulmonary immune cell trafficking promotes host defense against alcohol-associated Klebsiella pneumonia
Source: Commun Biol. 2021 Aug 23;4:997. doi: 10.1038/s42003-021-02524-0 (PMC8382828; doi:10.1038/s42003-021-02524-0)

Supplemental Methods:

Indole-metabolites detection by HPLC

Indole and its microbial metabolites were extracted with methanol. The cecal extracts were re-dissolved in 50% methanol for HPLC analysis (Waters, MA, USA) on a ZORBAX Eclipse XDB-C18 column (150 mm × 2.1 mm, 3.5 μm, Agilent Technologies, USA) at a flow rate of 0.25 mL/min. The linear gradient elution condition was: 20% mobile phase A (5% methanol/water with 0.1% formic acid) / 80% mobile phase B (100% methanol with 0.1% formic acid) for 3 min initially, then shifted to 50% B over 2 min and held at 50% B for an additional 10 min. The elution was monitored at UV 280 nm.

Supplemental Figure:

Supplemental Figure 1: Alcohol-feeding model characteristics Experiment 1. (A) Mouse weights in all groups at the start and end of the experimental period. (B) Average food intake in all groups. (C) Blood alcohol content of alcohol-fed mice with and without treatment. Bars represent the mean ± SEM. N = 10/group.

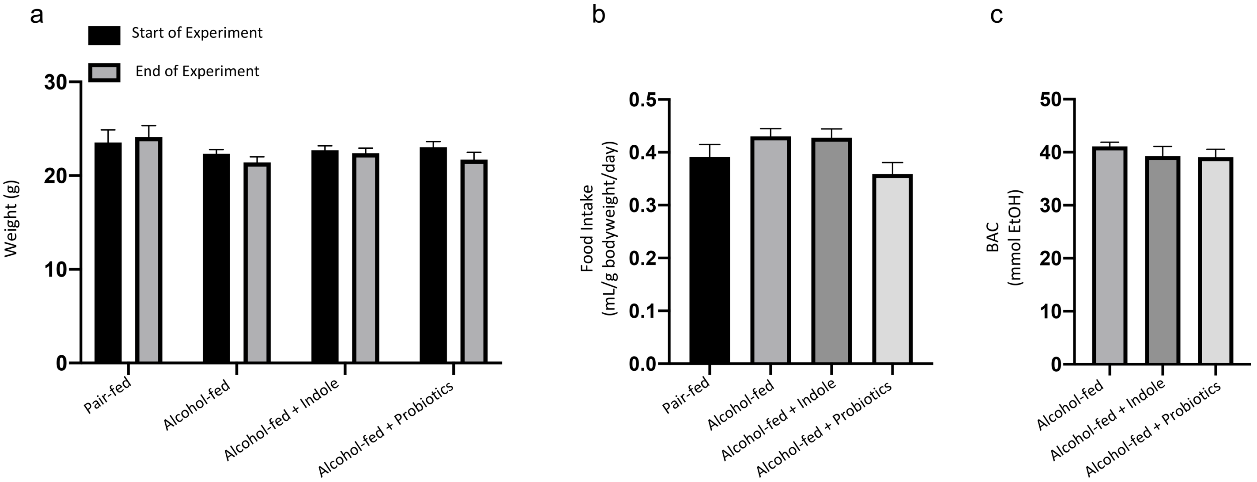

Supplemental Figure 2: Alcohol-feeding model characteristics Experiment 2. (A) Mouse weights in all groups at the start and end of the experimental period. (B) Average food intake in all groups. (C) Blood alcohol content of alcohol-fed mice with and without treatment. Bars represent the mean ± SEM. N = 10/group.

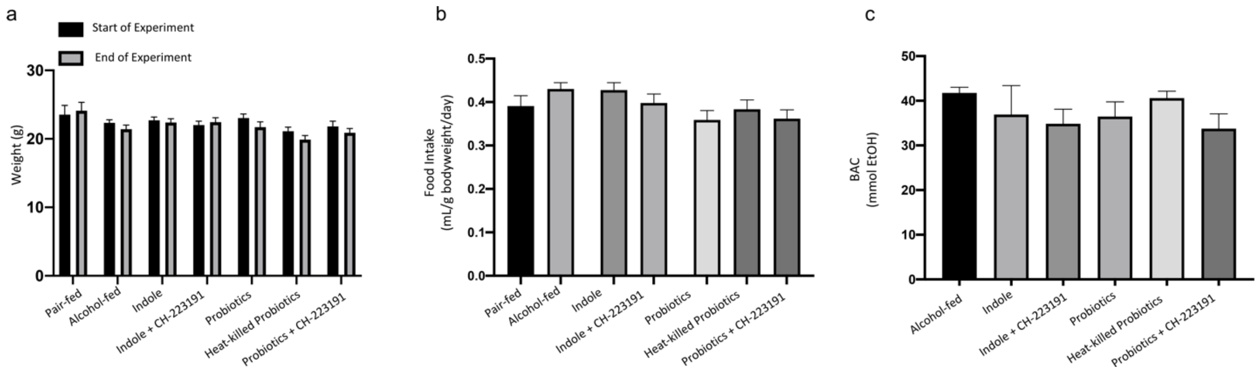

Supplemental Figure 3: Indole and probiotics alter intestinal indole metabolite concentrations of alcohol-fed mice. Binge-on-chronic alcohol-fed mice with and without treatment were infected with *Klebsiella* and sacrificed 48 hrs. post infection and the cecal concentrations of (a) indole-3-sulfate, (b) indole-3-acetic acid, (c) indole-3-lactic acid, and (d) indole-3-propionic acid were determined by HPLC. Bars represent the mean ± SEM and dots represent individual mice. P values are indicated in the figure and were determined by one-way ANOVA with Sidak's multiple comparison test. N = 5/group.

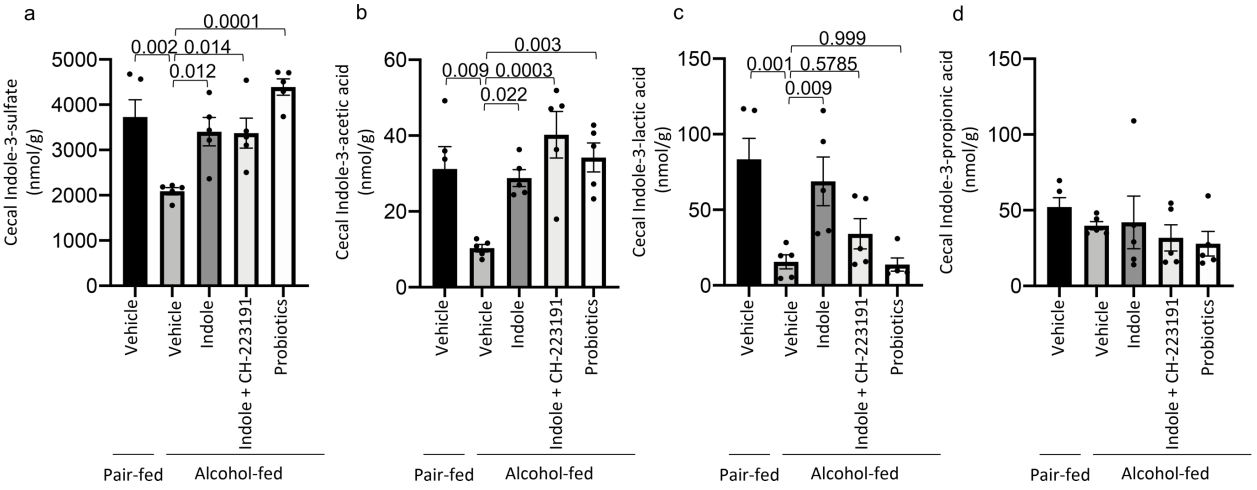

Supplemental Figure 4: TCDD increase AhR activation. DR-EcoScreen cells were treated with increasing concentrations of TCDD, to detect AhR agonistic activity. (a) Dose–response curves of TCDD in the DR-EcoScreen assay. (b) Indole's precent AhR agonistic activity compared to 10^{-10} M TCDD. Bars represent the mean ± SEM and dots represent individual mice. P values are indicated in the figure and were determined by one-way ANOVA with Sidak's multiple comparison test. N = 3/group.

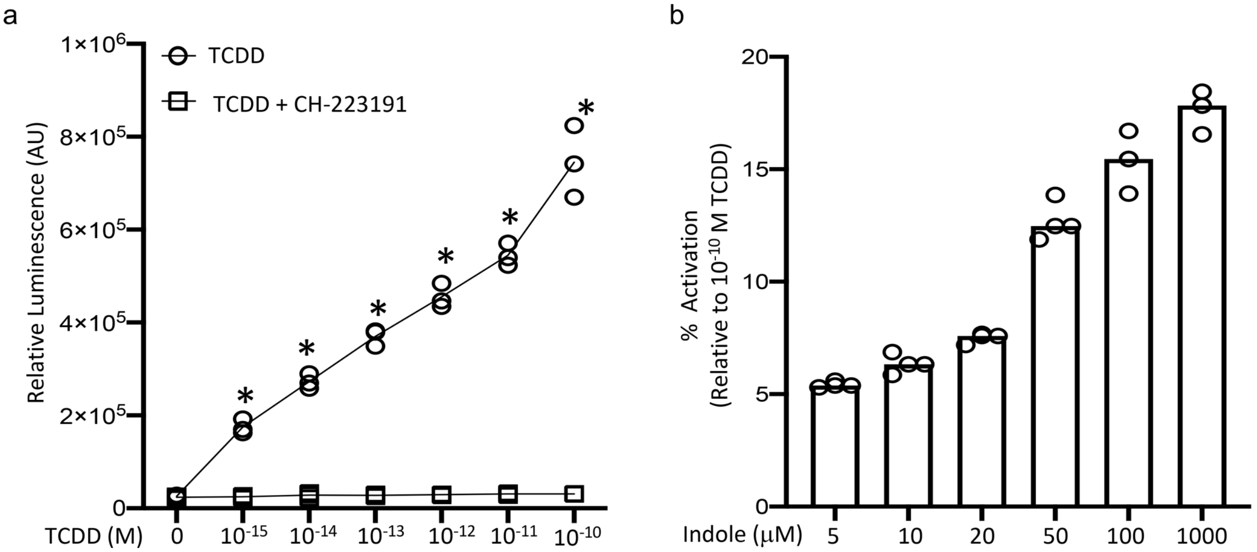

Supplemental Figure 5: Indole and probiotics alter the intestinal microbiota's inferred functional capacity. Binge-on-chronic alcohol-fed mice with and without treatment were infected with *Klebsiella* and sacrificed 48 hrs. post infection. (a) Principle coordinate analysis of the inferred functional capacities of the cecal microbiota from alcohol-fed mice with and without treatment. (b-e) Representative pathways and the relative differences between the significant groups, as determined using ANOVA followed by Tukey-Kramer post-hoc analysis with corrections for multiple comparison via FDR within the STAMP platform. N = 5/group.

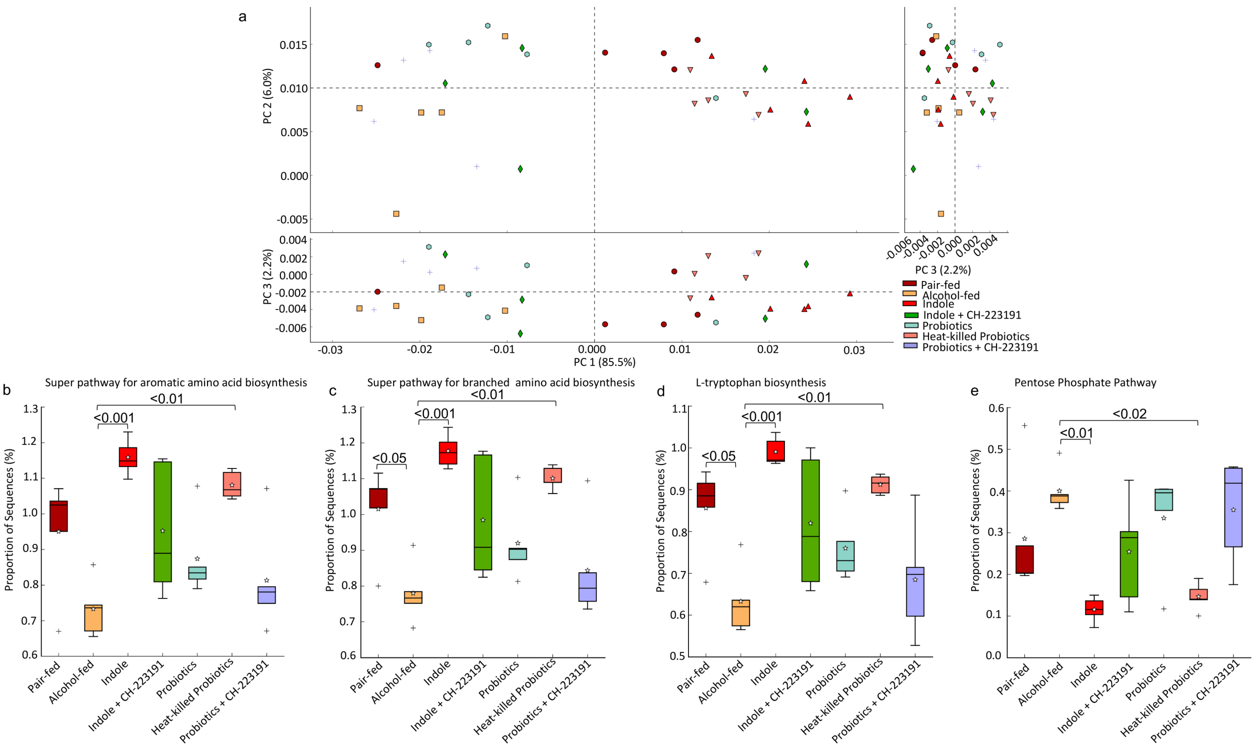

Supplemental Figure 6: Intestinal microbial structure and inferred functional capacity of alcohol-fed mice. (a) Abundance plots for *Lactobacillus reuteri*, and *Bifidobacterium animalis* in alcohol-fed and treated mice. Differentially abundant OTUs at the (b) phylum and (c) family level as determined by DESeq2. (b) Heat-map of significantly different metabolic pathways between groups of mice. N = 5/group.

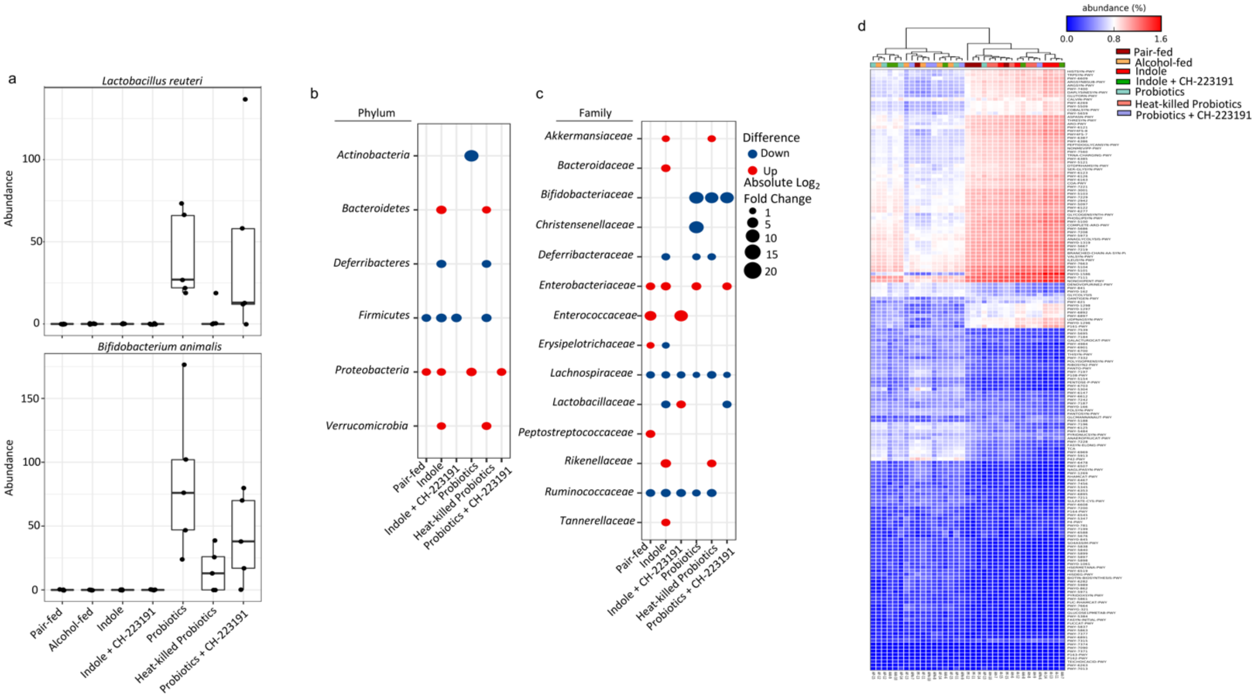

Supplemental Figure 7: Microbiota supplementation mitigates alcohol-associated extrapulmonary cell trafficking. The percentage of CFSE+ CD45+ immune cells in the (a) intestinal tract, (b) liver, and (c) spleen 48 hrs. post infection. Bars represent the mean ± SEM and dots represent individual mice. P values are indicated in the figure and were determined by one-way ANOVA with Sidak's multiple comparison test. N = 5/group.

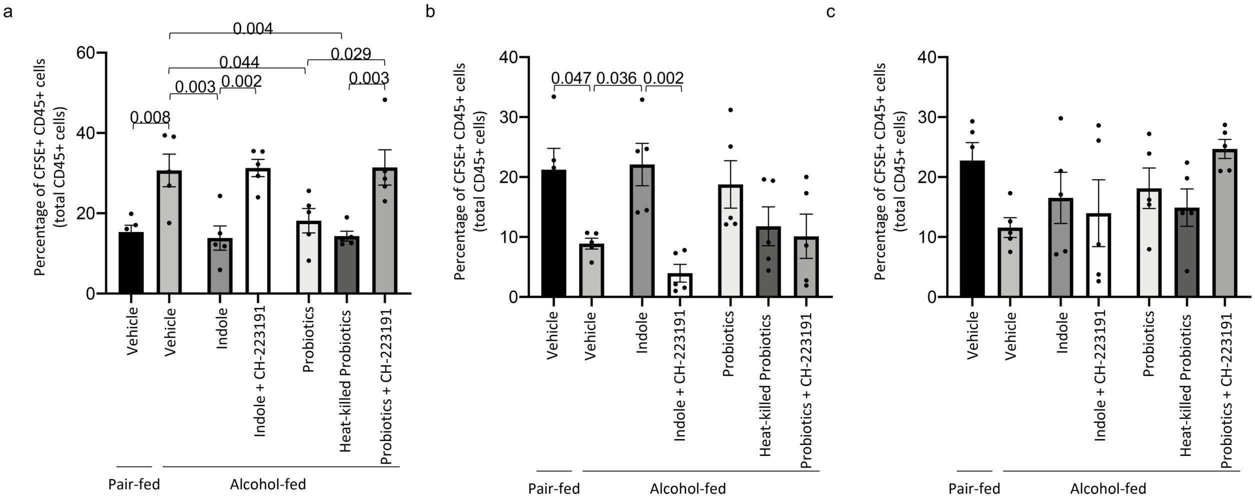

Supplemental Figure 8: CFSE histograms. Representative flow cytometry histograms from binge-on-chronic alcohol-fed mice with and without treatment in the (a) lungs, (b) intestine, (c) spleen, and (d) liver.

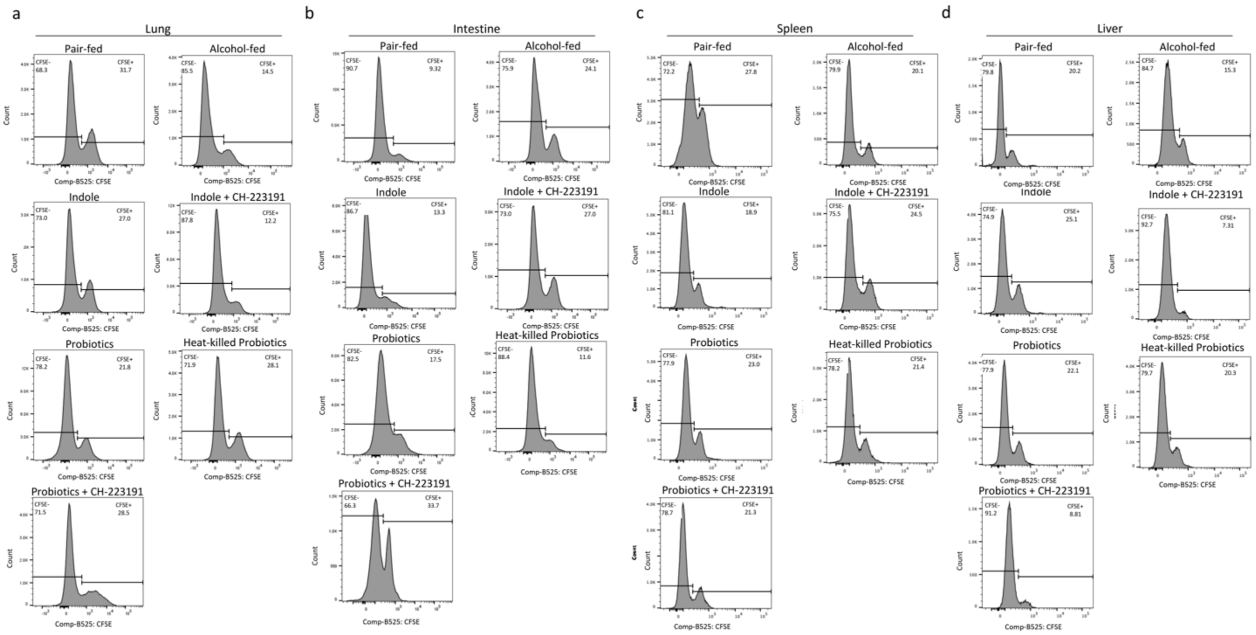

Supplement: Supplementary file 2 — Supplementary Information [file 42003_2021_2524_MOESM2_ESM.pdf]
