# Supplementary material for: Pulmonary immune cell trafficking promotes host defense against alcohol-associated Klebsiella pneumonia
Source: Commun Biol. 2021 Aug 23;4:997. doi: 10.1038/s42003-021-02524-0 (PMC8382828; doi:10.1038/s42003-021-02524-0)
Supplement: Supplementary file 3 — Description of Additional Supplementary Files [file 42003_2021_2524_MOESM3_ESM.pdf]

Description of Additional Supplementary Files

File name: Supplementary Data 1

Description: Excel file with all of the data for the figures and supplemental figures in their own labeled tab.
