# Supplementary material for: Pulmonary immune cell trafficking promotes host defense against alcohol-associated Klebsiella pneumonia
Source: Commun Biol. 2021 Aug 23;4:997. doi: 10.1038/s42003-021-02524-0 (PMC8382828; doi:10.1038/s42003-021-02524-0)
Supplement: Supplementary file 5 — Reporting Summary [file 42003_2021_2524_MOESM5_ESM.pdf]

nature research

Corresponding author(s): Derrick Samuelson

Last updated by author(s): Jul 6, 2021

Reporting Summary

Nature Research wishes to improve the reproducibility of the work that we publish. This form provides structure for consistency and transparency in reporting. For further information on Nature Research policies, see Authors & Referees and the Editorial Policy Checklist.

Statistics

For all statistical analyses, confirm that the following items are present in the figure legend, table legend, main text, or Methods section.

n/a	Confirmed	
☐	☒	The exact sample size (*n*) for each experimental group/condition, given as a discrete number and unit of measurement
☐	☒	A statement on whether measurements were taken from distinct samples or whether the same sample was measured repeatedly
☐	☒	The statistical test(s) used AND whether they are one- or two-sided<br>*Only common tests should be described solely by name; describe more complex techniques in the Methods section.*
☐	☒	A description of all covariates tested
☐	☒	A description of any assumptions or corrections, such as tests of normality and adjustment for multiple comparisons
☐	☒	A full description of the statistical parameters including central tendency (e.g. means) or other basic estimates (e.g. regression coefficient) AND variation (e.g. standard deviation) or associated estimates of uncertainty (e.g. confidence intervals)
☐	☒	For null hypothesis testing, the test statistic (e.g. *F*, *t*, *r*) with confidence intervals, effect sizes, degrees of freedom and *P* value noted<br>*Give P values as exact values whenever suitable.*
☒	☐	For Bayesian analysis, information on the choice of priors and Markov chain Monte Carlo settings
☒	☐	For hierarchical and complex designs, identification of the appropriate level for tests and full reporting of outcomes
☒	☐	Estimates of effect sizes (e.g. Cohen's *d*, Pearson's *r*), indicating how they were calculated

Our web collection on statistics for biologists contains articles on many of the points above.

Software and code

Policy information about availability of computer code

Data collection	No software was used for data collection.
Data analysis	Raw sequence data was processed using R and the R packages: DADA2 v1.1.5, Phyloseq v1.16.2, DESeq2 v1.20.0, PICRUSt2 v2.2.0, STAMP v2.1.3, and vegan v2.3-5

For manuscripts utilizing custom algorithms or software that are central to the research but not yet described in published literature, software must be made available to editors/reviewers. We strongly encourage code deposition in a community repository (e.g. GitHub). See the Nature Research guidelines for submitting code & software for further information.

Data

Policy information about availability of data

All manuscripts must include a data availability statement. This statement should provide the following information, where applicable:

- Accession codes, unique identifiers, or web links for publicly available datasets
- A list of figures that have associated raw data
- A description of any restrictions on data availability

Sequencing data is deposited in the National Center for Biotechnology Information Sequence Read Archive (BioProject ID: PRJNA602539). All additional data that support the findings of this study are available within the paper and its supplementary information files.

Field-specific reporting

Please select the one below that is the best fit for your research. If you are not sure, read the appropriate sections before making your selection.

☒ Life sciences ☐ Behavioural & social sciences ☐ Ecological, evolutionary & environmental sciences

For a reference copy of the document with all sections, see nature.com/documents/nr-reporting-summary-flat.pdf

Life sciences study design

All studies must disclose on these points even when the disclosure is negative.

Sample size	Sample size was calculated based on preliminary and published data of bacterial burden in alcohol fed mice.
Data exclusions	no data was excluded
Replication	Data was replicated
Randomization	All mice were acclimated to the institutional environment for 1 week. Mice were then randomly assigned to different treatment groups.
Blinding	No blinding was performed. Blinding was not possible as all researchers were need to perform the experiments and analysis the data, as no personal was available to setup each treatment or prepare samples for analysis so that the researchers were blinded.

Reporting for specific materials, systems and methods

We require information from authors about some types of materials, experimental systems and methods used in many studies. Here, indicate whether each material, system or method listed is relevant to your study. If you are not sure if a list item applies to your research, read the appropriate section before selecting a response.

Materials & experimental systems

n/a	Involved in the study
☐	☒ Antibodies
☐	☒ Eukaryotic cell lines
☒	☐ Palaeontology
☐	☒ Animals and other organisms
☒	☐ Human research participants
☒	☐ Clinical data

Methods

n/a	Involved in the study
☒	☐ ChIP-seq
☐	☒ Flow cytometry
☒	☐ MRI-based neuroimaging

Antibodies

Antibodies used	CD45, Biotin, BioLegend (103104) and Qdot 585, ThermoFisher (Q10111MP)<br>CD3, Alexa700, BioLegend (100216)<br>CD4, BUV 496 BD (564667)<br>CD8, BV711, BioLegend (100748)<br>CD11b, BV 510, BioLegend (101263)<br>CD11c, PerCP-Cy5.5, BioLegend (117328)<br>NK1.1, Alexa 647, BioLegend (108720)<br>Ly-6G/C (Gr1), PE-Dazzle 594, BioLegend (108452)<br>F4/80, BV 650, BioLegend (123149)
Validation	All antibodies were flow cytometry validated by the manufacture.

Eukaryotic cell lines

Policy information about cell lines

Cell line source(s)	Sekisui XenoTech via the JCRB Cell Bank
Authentication	validated by the manufacture.
Mycoplasma contamination	Tested by manufacture.
Commonly misidentified lines<br>(See ICLAC register)	*Name any commonly misidentified cell lines used in the study and provide a rationale for their use.*

Animals and other organisms

Policy information about studies involving animals; ARRIVE guidelines recommended for reporting animal research

Laboratory animals	Mouse, C57BL/6, female, age 10-12 weeks, obtained from Charles Rivers Labratories
Wild animals	NA
Field-collected samples	NA
Ethics oversight	Institute for Animal Care and Use Committee (IACUC) at LSUHSC and UNMC

Note that full information on the approval of the study protocol must also be provided in the manuscript.

Flow Cytometry

Plots

Confirm that:

☒ The axis labels state the marker and fluorochrome used (e.g. CD4-FITC).

☒ The axis scales are clearly visible. Include numbers along axes only for bottom left plot of group (a 'group' is an analysis of identical markers).

☒ All plots are contour plots with outliers or pseudocolor plots.

☒ A numerical value for number of cells or percentage (with statistics) is provided.

Methodology

Sample preparation	Iimmune cells were pretreated with TruStain FcX Anti-mouse CD16/32 antibody (BioLegend, San Diego, CA) and then stained with eFlour780 fixable viability dye (Invitrogen Eugene, OR) followed by staining with antibodies specific for murine CD45, CD3e, CD4, CD8a, GR1, NK1.1, CD11c, CD11b, and F4/80 (BioLegend).
Instrument	BD LSRII
Software	BD FACSDiva™ Software v9.0
Cell population abundance	No cell sorting was performed
Gating strategy	Gating strategy is defined within the paper.

☒ Tick this box to confirm that a figure exemplifying the gating strategy is provided in the Supplementary Information.
